# Genome-Wide Association Study Demonstrates the Role Played by the *CD226* Gene in Rasa Aragonesa Sheep Reproductive Seasonality

**DOI:** 10.3390/ani11041171

**Published:** 2021-04-19

**Authors:** Kenza Lakhssassi, Belén Lahoz, Pilar Sarto, Laura Pilar Iguácel, José Folch, José Luis Alabart, Malena Serrano, Jorge Hugo Calvo

**Affiliations:** 1 Centro de Investigación y Tecnología Agroalimentaria de Aragón, Instituto Agroalimentario de Aragón (IA2) (CITA–Zaragoza University), 50059 Zaragoza, Spain; klakhssassi@cita-aragon.es (K.L.); blahozc@cita-aragon.es (B.L.); mpsarto@aragon.es (P.S.); lpiguacel@cita-aragon.es (L.P.I.); jfolch@cita-aragon.es (J.F.); jlalabart@cita-aragon.es (J.L.A.); 2 INRA, Instituts Morocco, 6356 Rabat, Morocco; 3 Departamento de Mejora Genética Animal INIA, 28040 Madrid, Spain; malena@inia.es; 4 The Aragonese Foundation for Research and Development (ARAID), 50018 Zaragoza, Spain

**Keywords:** GWAS, reproduction, seasonality, oestrous, *CD226*, *NPY*

## Abstract

**Simple Summary:**

To elucidate the genetic basis of reproductive seasonality in Rasa Aragonesa sheep breed, we performed a genome-wide association study (GWAS) in order to detect single nucleotide polymorphisms (SNPs) or regions associated with traits related to ovarian function and behavioural signs of estrous. The GWAS included 205 ewes with genotypes for 583882 SNPs. Only one SNP overcame the genome-wide significance level. Nine potential SNPs overcame the chromosome-wise significance level (FDR 10%). Gene annotation demonstrated that *CD226* *molecule* (*CD226*) and *neuropeptide Y* (*NPY*) genes that could be involved in reproductive seasonality were close to the significant SNPs. To validate the results, we sequenced the entire coding region of the *NPY* gene and four exons of the *CD226* gene to search for polymorphisms that could be involved in the phenotypes studied. Two synonymous and two nonsynonymous SNPs in the *NPY* and *CD226* genes, respectively, were genotyped in the whole population. We demonstrated that the AA genotype of the SNP rs404360094 located in exon 3 of the *CD226* gene was associated with higher and lower total days of anoestrus and oestrous cycling months, respectively. Therefore, this SNP could be utilized as a genetic marker for assisted selection marker to reduce seasonality.

**Abstract:**

A genome-wide association study (GWAS) was used to identify genomic regions influencing seasonality reproduction traits in Rasa Aragonesa sheep. Three traits associated with either ovarian function based on blood progesterone levels (total days of anoestrus and progesterone cycling months) or behavioral signs of oestrous (oestrous cycling months) were studied. The GWAS included 205 ewes genotyped using the 50k and 680k Illumina Ovine Beadchips. Only one SNP associated with the progesterone cycling months overcame the genome-wide significance level (rs404991855). Nine SNPs exhibited significant associations at the chromosome level, being the SNPs rs404991855 and rs418191944, that are located in the *CD226 molecule* (*CD226*) gene, associated with the three traits. This gene is related to reproductive diseases. Two other SNPs were located close to the *neuropeptide Y* (*NPY*) gene, which is involved in circadian rhythms. To validate the GWAS, partial characterization of both genes by Sanger sequencing, and genotyping of two synonymous and two nonsynonymous SNPs in the *NPY* and *CD226* genes, respectively, were performed. SNP association analysis showed that only SNP rs404360094 in the exon 3 of the *CD226* gene, which produces an amino acid substitution from asparagine (uncharged polar) to aspartic acid (acidic), was associated with the three seasonality traits. Our results suggest that the *CD226* gene may be involved in the reproductive seasonality in Rasa Aragonesa.

## 1. Introduction

Sheep reproduction at temperate latitudes is widely known to exhibit marked seasonality [1]. The photoperiod represents a temporal signal to initiate changes in sheep reproductive status [2,3]. Lambing normally occurs at the end of winter-early spring, which causes seasonal variation in lamb production throughout the year. Accordingly, there is an imbalance between the availability of animal products and consumer demand. To overcome this disequilibrium, several alternatives have been developed, such as hormonal and/or photoperiod treatments. Nevertheless, at the initiative of the European Commission and to achieve “clean, green, and ethical” animal production [4], the use of hormones is being reviewed, primarily because it generates hormonal residues in animal carcasses. Therefore, breeders need other alternatives that minimize or completely avoid the use of hormonal treatments.

Differences in the duration of the breeding season between breeds and between individuals within a breed raised in the same region have been reported [5,6]. Ewes exhibiting spontaneous out-of-season ovulatory activity (SOA) are of considerable interest for identifying genes and mutations involved in molecular pathways controlling reproductive seasonality in sheep. In Rasa Aragonesa, Folch and Alabart [7] reported that approximately 25% of ewes have spontaneous ovulations in spring and can be naturally mated throughout the year with good management and feeding conditions. Therefore, developing a genetic approach for improving the out-of-season breeding ability of animals may represent a useful way to address this challenge. Hanocq et al. [8] reported relatively high heritability and repeatability estimates (0.20 and 0.30, respectively) for SOA in the Merinos d’Arles breed. Similar heritability of SOAs was found in the Chios breed in Greece [9] and in the Latxa breed in Spain [10]. Hence, the estimation of genetic parameters for SOA indicated that this trait could be used in selection [11].

Advances in genomic research and high-throughput genotyping techniques have enhanced the ability of researchers to search for mutations that underlie variations in complex traits [12,13]. Genome-wide association studies (GWAS) have become an important method for identifying genes and genomic regions associated with economically important traits in livestock. These studies are used to screen the whole genome for target genes that correlate with phenotypic traits using single nucleotide polymorphisms (SNPs) as genetic markers [14]. In sheep, progress has been made in the genetic basis of reproductive seasonality. In fact, three genes have been reported to be associated with out-of-season breeding: *melatonin receptor subtype 1A (MTNR1A)* [15,16,17,18,19,20,21,22,23,24,25], *arylalkylamine N-acetyltransferase (AANAT)* [26], and *leptin receptor (LEPR)* [27]. The first gene acts through high-affinity G-protein coupled receptors, one of which is melatonin receptor 1A encoded by the *MTNR1A* gene. Calvo et al. [28] and Lakhssassi et al. [27] showed that the SNPs rs403212791 in exon 2 of the *MTNR1A* gene and rs403578195 in exon 8 of the *LEPR* gene were associated with reproductive seasonality traits in the Rasa Aragonesa sheep breed. *AANAT* is involved in the biosynthesis of melatonin and controls daily changes in melatonin production. Additional candidate genes involved in seasonal breeding in sheep, such as the *aryl hydrocarbon receptor nuclear translocator-like protein (ARNTL)*, *casein kinase 1 epsilon (CSNK1E)*, *clock circadian regulator (CLOCK)*, *cryptochrome circadian regulator 1* (*CRY1)*, *period circadian regulator 1* (*PER1)*, *period circadian regulator 2* (*PER2)*, and *neuronal pas domain protein 4* (*NPAS4)*, have been reported in functional genomic studies of genes associated with circadian and circannual rhythms [29,30,31], although association studies of these genes with seasonality in ovine species have not been published to date.

As an initial attempt to identify genomic regions associated with reproductive seasonality in Rasa Aragonesa, our research group carried out a GWAS using 110 ewes genotyped by the Illumina OvineSNP50 Beadchip [32]. Several genes were identified near the significant SNPs, namely, *neuropeptide s receptor 1* (*NPSR1)*, *heparan sulfate-glucosamine 3-sulfotransferase 5* (*HS3ST5)*, *regulatory associated protein of mtor complex* 1 *(RPTOR)*, and *neuronal pentraxin 1* (*NPTX1)*, which are related to circadian and circannual rhythms. The aims of this study were the following: first, to perform a GWAS with a larger animal sample (205 ewes) using medium (50k) and high-density (680k) arrays from an Illumina Ovine Beadchip to identify new SNPs underlying sheep reproductive seasonality traits. Second, we confirmed the results obtained in the GWAS analysis through partial characterization of the genes located close to significant SNPs with putative functions related to seasonal reproduction and subsequent genotyping of some SNPs identified in these genes for association studies.

## 2. Materials and Methods

### 2.1. Animal Samples

Phenotypic seasonality data were collected from an experimental flock of Rasa Aragonesa sheep, described by Martinez-Royo et al. [32]. Flock management was the same for all ewes. Briefly, 3 different flock age groups were considered for a total number of 265 ewes: 155 mature ewes (5.2–7.2 y; 5.5 ± 0.5; mean ± SD), 84 young ewes (1.9 ± 0.0 y), and 26 lambs (0.94 ± 0.0 y). From these 265 ewes, 205 were used for GWAS distributed as follows: 122 mature (5.4 ± 0.45 y), 66 young (1.9 ± 0.0 y) and 17 ewe lambs (0.94 ± 0.0 y). Every three weeks, individual live weight (LW) and body condition score (BCS) on a 1 to 5 scale [33] were assessed. The mean LW and BCS were similar in the mature and young ewe age groups. The pooled overall means and standard deviations for mature and young ewes for the entire experimental period were 52.5 ± 7.7 kg and 2.9 ± 0.3 for LW and BCS, respectively. Ewe lambs had LW and BCS values of 40.6 ± 3.8 kg and 2.8 ± 0.1, respectively. The experimental period lasted from January to August 2012.

### 2.2. Measurement of Reproductive Seasonality Traits

In Martinez-Royo et al. [32] are reported the three reproductive seasonality phenotypes analysed in this study in detail. The first two phenotypes were based on weekly individual plasma progesterone level measurements. The total days of anoestrous (TDA) were the sum of days in anoestrous. This period of ovarian inactivity was characterised by three or more consecutive progesterone concentrations lower than a threshold of 0.5 ng/mL. The second reproductive seasonality phenotype was the progesterone cycling months (P4CM), defined for each ewe as the rate of cycling months according to the progesterone level measurements. Progesterone was determined using a commercial ELISA kit designed for ovine plasma (Ridgeway Science, St. Briavels, Gloucestershire, UK). An ewe was considered cyclic in a month when the progesterone concentration was higher than or equal to the threshold of 0.5 ng/mL in at least one progesterone determination in that month. It is outstanding that ewes with progesterone levels below the 0.5 ng/mL threshold in all samples taken in January and with more than 4 consecutive samples higher than the threshold (potential pathological ewes) were discarded for the GWAS. In the same way, the ewes had to be cycling in the preceding breeding season, according to three progesterone determinations performed one week apart in October. Finally, the oestrous cycling months (OCM) was the third phenotype analysed. This trait was explained for each ewe as the rate of months cycling based on daily oestrous records, using eight vasectomised rams fitted with breeding harnesses with a replaceable marking crayon [34]. Consequently, oestrous was recorded daily as crayon marks on the rumps of the ewes.

### 2.3. Sampling and Genotyping Analysis

The GWAS included 110 ewes genotyped with the OvineSNP50 Infinium Beadchip (Illumina Inc., San Diego, CA, USA) designed by the International Sheep Genome Consortium [35], as employed in the study performed by Martinez-Royo et al. [32], and 97 ewes genotyped with the 680k (IlluminaAgResearchSheep HD). Genomic DNA was extracted from blood samples using the SpeedTools DNA Extraction kit (Biotools, Madrid, Spain). SNP genotyping services were provided by the Spanish centre “Centro Nacional de Genotipado (CEGEN-ISCIII)” (https://www.usc.es/cegen/; accessed on 19 April 2021) and “Xenetica Fontao” company (https://www.xeneticafontao.com; accessed on 19 April 2021).

### 2.4. Data Quality Control and Genome-Wide Association Analysis

The software Plink1.9 [36] was used for quality control (QC) of each genotyped data. Individuals with a low call rate (< 0.90) were excluded from additional analysis. The SNPs that met the following criteria were selected: call rate > 0.97 and minor allele frequency (MAF) > 0.01. SNPs that failed Hardy–Weinberg equilibrium (HWE) (*p*-Value < 0.001) were excluded. Next, the two datasets were merged with PLINK 1.9, and the Beagle4.0 program [37] was used to impute 50k to 680k genotypes. We performed clustering and multidimensional scaling (MDS) to check for population stratification using PLINK 1.9. SNPs that passed QC were pruned using the linkage disequilibrium (LD) pruning parameters of r^2^ < 0.2 over a window size of 50 SNPs, and a step of 10 SNPs. Genome-wide identity-by-state (IBS) pairwise distances were calculated using all SNPs that remained after pruning. A pairwise population concordance test constraint was applied to the clustering procedure (-ppc option). GWAS was performed using the GCTA (Genome-wide Complex Trait Analysis) program [38] running a mixed linear model association (MLMA) and excluding the chromosome on which the candidate SNP is located (leaving-one-chromosome-out, or LOCO). The model also considered age and clusters obtained by MDS as a fixed effect, and BCS and LW effects as quantitative covariates. The estimated genetic relationships matrix (GRM) was included in the mixed model analysis to correct the effect of population substructure. The significance of association was assessed using Bonferroni correction and the false discovery rate (FDR) multitest correction tests. Chromosome-wise significance association was also assessed using a false discovery rate (FDR = 0.1) multitest correction threshold [39]. The choice of a threshold of 10% can be explained by the fact that in this study, the objective is mainly exploratory in order to identify new SNPs underlying sheep reproductive seasonality traits, and these are low heritable female traits. Visualization of the association data in Manhattan plots and quantile-quantile plots was performed using SNPEVG software [40]. To control the number of false positives, genomic inflation factors were calculated in R v3.5.1 software for each reproductive seasonality trait. The genomic inflation factor was estimated as the observed median χ^2^ divided by the expected median χ^2^.

### 2.5. Gene Identification

Genes located within a 500 kb-long interval centered on the significant SNPs associated with the three seasonality traits were obtained according to the sheep genome assembly (Oar_v3.1) and based on Ensembl release 81.

### 2.6. Validation of GWAS Results

#### 2.6.1. CD226 and NPY Gene Characterization

Gene annotation based on the results of GWAS demonstrated that *CD226* and *NPY* genes could be involved in reproductive seasonality. The ovine *CD226* gene is located on chromosome 23, covering approximately 100.7 kb with 6 exons (GenBank acc. Number NC_040274), whereas the *NPY* gene is located on chromosome OAR4 and covers approximately 6.8 kb with 4 exons (GenBank acc. Number NC_040255). All exons of the *NPY* gene were characterized, while four exons were chosen to characterize the *CD226* gene. The significant SNPs were located in intron 2 of *CD226*, approximately 52 and 4 kb from exons 2 and 3, respectively. These exons were selected because they have nonsynonymous polymorphisms in the Ensembl Variation database (https://www.ensembl.org/info/genome/variation/index.html; accessed on 19 April 2021) of the Oar 3.1 version of the sheep genome. Primer3 software was used to design the primers (https://bioinfo.ut.ee/primer3–0.4.0/; accessed on 19 April 2021). Target-specific primers were designed in intron-flanking regions around the targeted exon. Table 1 shows the oligonucleotide sequences, the amplified exon, the annealing temperature, and expected product sizes. Genomic DNA was extracted from blood samples using standard protocols. The genomic DNA (25 ng) was amplified in a final polymerase chain reaction (PCR) volume of 25 µL containing 5 pmol of each primer, 200 nM dNTPs, 2.4 mM MgCl_2_, 50 mM KCl, 10 mM Tris-HCl, 0.1% Triton X-100, and 1 U Taq polymerase (Biotools, Madrid, Spain). The following cycling conditions were used for all amplification fragments: an initial denaturation step of 94 °C for 3 min followed by 35 cycles of PCR, with cycling conditions of 30 s at 94 °C, 30 s at annealing temperature, and 30 s at 72 °C, and a final extension step of 72 °C for 5 min.

Direct Sanger sequencing of the PCR products from the 8 exons in 18 ewes with extreme and intermediate values for TDA (low TDA: 0 days, *n* = 6; intermediate TDA: 56 ± 19.8 days, *n* = 6; high TDA: 142.3 ± 15.7 days, *n* = 6) was utilized to look for polymorphisms in the experimental population. The PCR products were purified using the FavorPrep Gel/PCR purification mini kit (Favorgen, Ibian, Zaragoza, Spain), and sequenced in both directions by STAB Vida company (Caparica, Portugal) using an ABI 3730XL sequencer (Applied Biosystems, Foster City, CA, USA). Homology searches were performed using BLAST (National Centre for Biotechnology Information: https://blast.ncbi.nlm.nih.gov/Blast.cgi; accessed on 19 April 2021). To align the sequences, BioEdit [41] software and CLUSTAL Omega (http://www.ebi.ac.uk/Tools/msa/clustalo/; accessed on 19 April 2021) were used. The impact of amino acid substitution on the structure and function of the protein was predicted using the PolyPhen-2 (http://genetics.bwh.harvard.edu/pph2/; accessed on 19 April 2021) [42] and Variant Effect Predictor (VEP: http://www.ensembl.org/Ovisaries/Tools/VEP?db=core; accessed on 19 April 2021) softwares. The locations of SNPs were determined based on the genome version of *Ovis aries* Oar_v3.1.

#### 2.6.2. CD226 and NPY Polymorphism Genotyping

Genomic DNA was extracted from blood samples of 265 ewes of the flock using standard protocols. Two nonsynonymous SNPs in the *CD226* gene were selected for genotyping the whole population by Kompetitive allele-specific PCR (KASP) following the manufacturer’s instructions: one in exon 2 (rs588529642) and the second in exon 3 (rs404360094). The sequence surrounding the target polymorphism was submitted for assay design to the KASP genotyping provider (LGC Genomics, Biotools, Spain). Assays were performed in 96-well formats in a 10 μL volume containing 1 µL of DNA (20 ng final concentration of DNA), 5 µL of 2X KASP V4.0 master mix standard ROX (LGC Genomics, UK), 0.14 µL of assay mix (KASP by Design assay mix, LGC Genomics, UK) and 3.86 µL nuclease-free water. Reactions were performed in a CFX96 Bio-Rad thermocycler (Bio-Rad, Madrid, Spain) with conditions as follows: 94 °C for 15 min followed by 9 touchdown cycles of 94 °C for 20 s and 57 °C for 60 s (decreasing −0.6 °C per cycle) followed by 25 additional cycles of 20 s at 94 °C and 60 s at 55 °C. Following PCR, the plate was cooled to 30 °C, and fluorescence was read using a single quantification cycle for 1 s.

The assay designed to genotype the *NPY* gene failed. Therefore, the two synonymous SNPs located in exon 2 of the *NPY* gene (OAR4: g.71593018G > T, and rs594346709) were genotyped by Sanger sequencing (as described above in Section 2.6.1).

#### 2.6.3. SNP Association Studies

Haploview software v4.2 [43] was used to calculate the Hardy–Weinberg equilibrium exact test, the observed and expected heterozygosities and the minor allele frequency (MAF) for each SNP. Statistical association studies between the SNPs and reproductive seasonality traits (TDA, P4CM, and OCM) were performed by fitting a linear model using the Rcmdr package of R software (http://socserv.socsci.mcmaster.ca/jfox/Misc/Rcmdr/; accessed on 19 April 2021) [44], including the genotype of the SNPs (S) and age (mature, young and ewe lambs) (A) as fixed effects. The LW and (CS were fitted as covariates. The least square means (LSMs) for each pairwise comparison were calculated to test differences between genotypes. The Bonferroni correction was used to consider multiple tests. The *CD226* and *NPY* SNPs were independently analysed with the same statistical model.

#### 2.6.4. Haplotype Association Studies

Haploview software v4.2 was used to define blocks of LD based on the 4-gamete rule [43]. D’ and r^2^ within the *CD226* and *NPY* were estimated. SNPs were phased using the expectation-maximization (E–M) algorithm to assign individual haplotypes with PLINK1.9 [45]. Diplotypes with a posterior probability lower than 0.7 were discarded. Haplotype association studies were performed using the Rcmdr package by fitting a similar lineal model to that used for the SNP association studies but included the haplotype (H) effect instead of the S effect. The number of copies of each haplotype were codified as 0, 1, or 2 copies. Haplotypes with <1% frequency were not included in the analysis. The LSMs for each pairwise comparison were calculated to test differences between haplotypes. The Bonferroni correction was used to consider multiple tests.

## 3. Results

### 3.1. GWAS Results

After the QC was performed on the imputed genotypes, 205 ewes with genotypes for 583882 SNPs distributed on the 27 ovine chromosomes were retained for subsequent analyses. In the MDS analysis, 188633 autosomal SNPs were used to calculate the pairwise IBS distance after SNP pruning. MDS analysis revealed a substructure within the total dataset and identified 4 principal clusters in the analysed population (Figure S1). These clusters were taken into account for subsequent association analysis. The GWAS results obtained through MLMA and LOCO were very similar. The genomic inflation factors for each trait were less than 1 (TDA: 0.96; P4CM: 0.97; OCM: 0.95), and then no more adjustment was needed.

**Figure 1:**
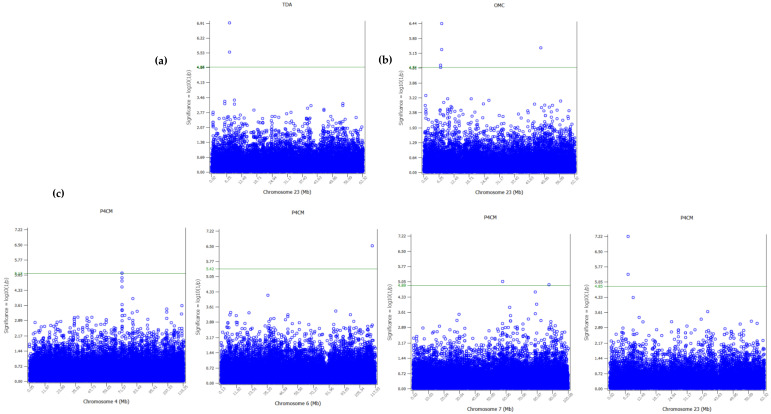
Manhattan plots of chromosome-wise association studies of (**a**) total days of anoestrous (TDA), (**b**) oestrous cycling month (OCM) and (**c**) progesterone cycling month (P4CM) traits. Only chromosomes with significant SNPs are shown. The horizontal green line in Manhattan plots corresponds to the average threshold value for an FDR of 10% evaluated at the chromosomal level.

Only one significant SNP (rs404991855) for P4CM was found at the genome-wise significance level after Bonferroni correction (Table S1). In the same way, a trend was observed for the same SNP and TDA trait (*p* = 0.07). Figure S2 presents the Manhattan and Q-Q plots for the three traits. Furthermore, 2, 7, and 4 SNPs were significantly associated at the chromosome-wise level (FDR *p* < 0.10) with TDA, P4CM, and OCM traits, respectively (Figure 1 and Table 2). Two SNPs (rs404991855 and rs418191944) located on chromosome 23 were found to be significantly associated with the three traits at the chromosome level (Figure 1 and Table 2). The seven SNPs associated with P4CM variability were located on chromosomes 4 (rs424340754, rs410373132), 6 (rs409834034), 7 (rs428238419 and rs405959180), and 23 (rs404991855 and rs418191944) (Figure 1c). Finally, four SNPs on chromosome 23 (rs405024177, rs404991855, rs418191944 and rs410842314) were associated with OCM (Figure 1b). The genes located 250 kb upstream and downstream of the most significant SNPs for each seasonality trait are shown in Table 2. Two genes were of interest because they may be related to reproductive seasonality trait variation. The *NPY* gene, and *CD226* gene that is also known as *DNAM-1* (*DNAX Accessory Molecule-1*). The significant SNPs for P4CM in chromosome 4 were located 35 (rs424340754) and 47 kb (rs410373132) from *NPY* and were completely linked. The SNPs rs404991855 and rs418191944 were located in intron 2 of *CD226*.

### 3.2. Validation Studies

We sequenced the entire coding region of the *NPY* gene and four exons of the *CD226* gene to search for polymorphisms that could be involved in the phenotypes studied. No deleterious mutations were detected. For the *NPY* gene, we found two synonymous SNPs in exon 2. One of these mutations was not previously described (OAR4:g.71593018 G > T) (Table 3). These SNPs showed low MAFs, ranging between 0.025 (g.71593018 G > T) and 0.082 (rs594346709) (Table S2). Although these mutations were synonymous, they were genotyped in the whole population, since they might be in linkage disequilibrium with putative causative mutations.

For the *CD226* gene, three SNPs were detected in exon 2 (rs427511555, rs403900117 and rs588529642), and one SNP was detected in exon 3 (rs404360094). Two of these SNPs (rs588529642 and rs404360094) were nonsynonymous substitutions and were selected to be genotyped in the whole population. These SNPs were predicted in silico to be tolerated by VEP and benign by PolyPhen-2, showing that the SNP located in exon 2 (rs588529642) had a lower SIFT value (0.1) (Table 3). This SNP produces a change of methionine to valine (Met60Val), both of which belong to hydrophobic groups (nonpolar). Conversely, the SNP located in exon 3 (rs404360094) produces an amino acid substitution from asparagine (uncharged polar) to aspartic acid (acidic) at position 243.

To determine the extent of LD among these markers, we estimated the parameters D’ and r^2^ between the pairwise combination of the SNPs in the same chromosome. Very low LD was found. LD indices for SNPs located in the *CD226* gene were 0.06 and 0 for D’ and r^2^, respectively. For SNPs located in the *NPY* gene, 1 and 0.002 were observed for D’ and r^2^, respectively. One haplotype block was predicted for SNPs located in exon 2 of the *NPY* gene.

### 3.3. SNP Association Studies

Association analyses included 265 ewes. Table 4 shows results after Bonferroni correction. Only the SNP located in exon 3 of the *CD226* gene was significantly associated with the three reproductive seasonality traits (see Table S3 for further details). In fact, significant differences were observed among alternative genotypes. Ewes with the AA genotypes had higher TDA values than ewes with AG (50.80 ± 12.76; *p* < 0.01) or GG (42.52 ± 12.81; *p* < 0.05) genotypes. Similarly, ewes with the AA genotype showed fewer oestrous records than those with AG (−0.17 ± 0.05; *p* < 0.01) or GG (−0.13 ± 0.05). Similar results were observed for the P4CM trait.

### 3.4. Haplotype Association Studies

Haplotype association studies were performed, considering block 1, which was predicted with Haploview (the two SNPs in exon 2 of the *NPY* gene), and a second block containing the two nonsynonymous SNPs of the *CD226* gene (block 2). Three haplotypes were identified for each block (Table S4). Only diplotypes with a posterior probability higher than 0.7 and haplotype frequency > 1% were considered. The results of haplotype analysis are presented in detail in Tables S5 and S6 for blocks 1 and 2, respectively. After Bonferroni correction, only haplotypes H1 and H2 of block 2 were associated with the three reproductive seasonality traits, thereby confirming the previous association found in SNP analysis (Table 5). In this sense, ewes with one or 2 copies of haplotype H1 (A**G**) had lower TDA and more oestrous records than those without copies (in bold the SNP allele associated with the trait). This haplotype contains the G allele of the SNP located in exon 2 (rs418191944), which is associated with lower TDA and higher P4MC and OCM values. Conversely, ewes with 2 copies of haplotype H2 (A**A**) had more TDA and fewer oestrous records.

## 4. Discussion

Nine potential SNPs reaching the chromosome-wise level of significance were identified to be associated with the TDA, P4CM, and OCM reproductive seasonality traits. Only one SNP (rs404991855) associated with P4CM overcame the genome-wide significance level. The SNPs rs404991855 and rs418191944 on chromosome 23 appeared to be associated with the three traits and were located in intron 2 of the *CD226* gene. This gene has been related to such reproductive diseases as cystic ovarian teratoma and mature teratoma of the ovary in humans [46,47]. On the other hand, SNPs (rs424340754 and rs410373132) in chromosome 4 were significantly associated with P4CM. These SNPs are located in an intergenic region approximately 34 kb from the *NPY* gene, which influences many physiological processes, including circadian rhythms, anorexia, and weight loss [48]. Three other SNPs (rs409834034, rs428238419, and rs405959180) in chromosomes 6 and 7 were also significantly associated with this trait. However, none of the genes annotated in the 250 kb region around the significant SNPs was related to reproductive traits. Finally, four SNPs located in chromosome 23 were associated with the OCM trait: two in intron 2 of the *CD226* gene (rs404991855 and rs418191944), one in an intergenic region approximately 412 kb from the *CD226* gene (rs405024177), and one in a different intergenic region in chromosome 23 (rs410842314).

The *NPY* and *CD226* genes have been reported in other studies to be potentially associated with reproductive processes. *NPY* plays a role in the regulation of the secretion of gonadotropin releasing hormone (GnRH) in rats [49], rabbits [50], monkeys [51], and sheep [52,53]. It has been shown that NPY-containing neurons in the hypothalamus hold oestrogen receptors in rats [54]. In the same way, Skinner and Herbison [55] indicated that changes in photoperiod may regulate oestrogen receptor expression within the preoptic area. These authors suggested that hypothalamic NPY and β-endorphin neurons are involved in the seasonal regulation of ewe reproductive activity. Barker-Gibb and Clarke [56] reported a reduction in the number of NPY cells detectable by immunohistochemistry in the nonbreeding season compared to the breeding season in ovariectomized ewes either treated or not treated with oestrogen. Williams et al. [57] noted that NPY and other unidentified neurons in the hypothalamus interact with leptin, which is an important mediator of energy homeostasis and reproductive status in sheep. In pigs, NPY modulates hypothalamic neuronal activity (LH secretion) and serves as a putative link between the metabolic state and the reproductive axis [58]. Clarke et al. [48] determined that *NPY* stimulates feed consumption and inhibits reproduction in sheep. However, Miner [59] showed that NPY is a potent orexigenic agent in sheep, as in other species, and a seasonal change in the expression of this peptide may be related to stimulation of feed intake, rather than seasonality. On the other hand, the *CD226* gene encodes the glycoprotein CD226, also known as DNAM-1 (DNAX Accessory Molecule-1), which is expressed on the surface of natural killer (NK) cells and is involved in T cell-mediated cytotoxicity against certain tumours [60]. NK cells express a repertoire of activating and inhibitory receptors that control NK cell cytotoxicity and interferon-γ (IFNG) production to ensure self-tolerance while allowing efficacy against such insults as viral infection and tumour development [61,62]. NK cells are also known to play an important role in human reproduction. These cells can be categorized into two main populations based on the relative expression of the surface markers CD^16^ and CD^56^: CD^56bright^/CD^16–^ functioning to primarily produce cytokines in the circulating blood, and CD^56dim^/CD^16+^ performing cytotoxicity in the tissues [63,64]. Lukassen et al. [65] have shown that the proportion of CD^56dim^/CD^16+^ NK cells in follicular fluid (FF) in women suffering from idiopathic infertility is significantly higher than that in FF from patients undergoing in vitro fertilization (IVF) for tubal or male factor infertility. Furthermore, Křížan et al. [66] reported that the FF of patients with successful IVF outcomes was enriched with CD^56bright^/CD^16–^ NK cells. Additionally, Fainaru et al. [67] indicated that CD^56bright^ CD^16–^ NK cells are abundant in maturing ovarian follicles and that their presence correlates with the ovarian response to gonadotropins. These cells have been shown to be proangiogenic through the secretion of such cytokines as vascular endothelial growth factor and placental growth factor [68]. In patients with a good response to ovarian stimulation, CD^56bright^ NK cells migrate into the ovarian follicle, supporting follicular angiogenesis and oocyte development [67]. Recent research conducted by Stannard et al. [46] described a new CD^56dim^ NK cell subset characterized by a lack of expression of DNAM-1. These researchers reported that CD^56dim^DNAM-1^neg^ NK cells displayed reduced motility, poor proliferation, lower production of interferon-γ, and limited killing capacities compared with CD^56bright^ and CD^56dim^DNAM-1^pos^ NK cell subsets.

According to the aforementioned findings, it can be inferred that mutations in *CD226* could lead to glycoprotein alteration and could therefore affect follicle development. Additionally, mutations in the *NPY* gene would explain one of the neuroendocrine and physiological mechanisms that link nutrition and reproduction. Accordingly, the *NPY* and *CD226* genes were selected for validation studies for their possible involvement in seasonal reproduction in sheep. Using 18 ewes with extreme values of TDA, we detected 2 and 4 SNPs in *NPY* and *CD226*, respectively (Table 3). This design could increase the power to detect polymorphic SNPs associated with the trait but minimize the probability of detecting other polymorphic SNPs.

In this study, no association was found with the *NPY* gene. These SNPs show a low MAF (0.025 and 0.08 for OAR4: g.71593018 G > T and rs594346709, respectively). Notably, we did not sequence regulatory regions, such as the promoter or 5’ and 3’ UTR regions. Therefore, we could have missed the responsible mutation, as it was not in LD with the detected mutations. Similarly, no association was found with the SNP located in exon 2 of the *CD226* gene. Importantly, no homozygous animals were found for the G allele, and a low MAF was found for this SNP (0.042). However, the analysis demonstrated that the SNP located in exon 3 of *CD226* (rs404360094) was associated with the three reproductive seasonality traits. Notably, this SNP is close and in LD with the significant SNPs found in intron 2 in the GWAS analysis (4 and 4.7 kb from SNPs rs418191944 and rs404991855, respectively). In this sense, the GWAS analysis could have detected the effect found in exon 3 of *CD226*. Heterozygous and homozygous ewes for the G allele had less TDA and showed more oestrous events than those with the AA genotype (Table 4), which were observed at low frequency (4.7%) in our sample (Table S2). These results were confirmed by haplotype analysis such that animals carrying 1 or 2 copies of the H1 haplotype (containing the G allele) showed less TDA and higher P4CM and OCM values. Similarly, animals with two copies of the H2 haplotype (containing the A allele) showed higher TDA and lower P4CM and OCM values. This SNP produces an amino acid substitution from asparagine (uncharged polar) to aspartic acid (acidic) at position 243 at the end of the second immunoglobulin V-like domain in the extracellular region of the protein and close to the transmembrane motif [69]. However, the real effect of this variant is not clear because it has been predicted in silico to be a tolerant nonsynonymous substitution. It should be noted that we did not sequence the complete coding or regulatory regions of the *CD226* gene; thus, the observed relationship could indicate that the SNP should be in LD with the true causative mutation.

In summary, GWAS results were confirmed using a candidate gene approach. First, two SNPs located in intron 2 of the *CD226* gene reached the chromosome-wise significance threshold on chromosome 23 using the GWAS approach. As noted above, because this gene could be related to follicular angiogenesis and oocyte development in humans, we decided to confirm the GWAS results using a candidate gene approach, determining that the SNP rs404360094 located in exon 3 of the *CD226* gene was associated with reproductive seasonality traits in Rasa Aragonesa ewes. Therefore, this SNP could be utilized as a genetic marker for assisted selection to reduce seasonality. These results should be confirmed in more animals and in other breeds.

## 5. Conclusions

This study employed a GWAS approach to identify genomic regions associated with traits involved in reproductive seasonality in sheep. We demonstrated that the G allele of the SNP rs404360094 located in exon 3 of the *CD226* gene is associated with lower TDA and higher P4CM, which are both traits related to ovarian function based on blood progesterone levels. This allele was also associated with higher OCM scores, which is an indicator of oestrous behaviour. These findings enabled us to validate the GWAS results and demonstrated the involvement of the genomic region where the *CD226* gene is located. This SNP could be utilized as a genetic marker along with other SNPs already characterized in Rasa Aragonesa as being associated with traits related to reproductive efficiency, such as prolificacy or reproductive seasonality.

## Figures and Tables

**Table 1:** Primer sequences, amplified exon (site), annealing temperatures (AT), and amplification fragment sizes.

Target Gene	Site	Primer Sequence (5′–3′) ^1^	AT (°C) ^2^	Size (bp)
*CD226*	Exon 1	F: GCATGATGGCAAGGATTTTT	52	486
	-	R: GCGTCATAAATTCTGAACGTG	-	-
	Exon 2	F: TTTCTGATATTTCTCTGGTGTTTCA	52	493
	-	R: GACCCCAAAATGGGATAAGG	-	-
	Exon 3	F: CCTCATATCCAAGAACTTGAGGA	52	498
	-	R:TGTATAAGAAAGTCATGAGAAAGACAA	-	-
	Exon 4	F: TCCCAACTTCCTCTCTATTCTAGC	55	212
	-	R: GCATCAGAATTACTCAGGAGGAG	-	-
*NPY*	Exon 1	F: CACAGGGGTTAGGGATCG	55	236
	-	R: AGCCATAAAAACCCTGTTGC	-	-
	Exon 2	F: AAGATGCCCATGATCTCCAG	55	300
	-	R: GAATTCCCTAAGCCCCCTTC	-	-
	Exon 3	F: CTTTCCCTGACCACCTTGAG	55	188
	-	R: AAGAACTTTTACTCCCCCAACC	-	-
	Exon 4	F: TGACGACAAAGGGAAACTGC	55	220
	-	R: TCTTACAAGCCTCCCAGGAA	-	-

^1^ F: forward; R: reverse; ^2^ AT: annealing temperature.

**Table 2:** Significant SNPs at the chromosome-wise level associated with each seasonality trait. The SNPs are ordered according to their positions in the Oar 3.1 genome version (Ensembl release 81). Minor allele frequency (MAF) is also indicated. Putative causal genes located in the 250 kb region on both sides of the significant SNPs are indicated.

Trait	SNP	dbSNP	Chr	Position	MAF	*p*-Value	Genes within 250 kb on either Side
TDA	oar3_OAR23_7427625	rs404991855	23	7427625	0.40	1.22 × 10^−7^	*RTTN* -*CD226*-DOK6
	oar3_OAR23_7428353	rs418191944	23	7428353	0.39	2.77 × 10^−6^	*RTTN -CD226-DOK6*
P4CM	oar3_OAR4_71540823	rs424340754	4	71540823	0.22	7.12 × 10^−6^	*NPY*
	oar3_OAR4_71552651	rs410373132	4	71552651	0.22	7.12 × 10^−6^	*NPY*
	oar3_OAR6_114690755	rs409834034	6	114690755	0.21	3.01 × 10^−7^	*ENSOARG00000011847-ENSOARG00000013314-LRPAP1- ENSOARG00000013472-ENSOARG00000013494 -ENSOARG00000013502- RGS12*
	oar3_OAR7_57807908	rs428238419	7	57807908	0.04	8.59 × 10^−6^	*DTWD1- ENSOARG00000020999- FGF7- GALK2*
	oar3_OAR7_87670575	rs405959180	7	87670575	0.12	1.22 × 10^−5^	*ENSOARG00000002769*
	oar3_OAR23_7427625	rs404991855	23	7427625	0.40	6.07 × 10^−8^	*RTTN -CD226-DOK6*
	oar3_OAR23_7428353	rs418191944	23	7428353	0.39	3.83 × 10^−6^	*RTTN -CD226-DOK6*
OCM	oar3_OAR23_6962033	rs405024177	23	6962033	0.08	2.30 × 10^−5^	*SOCS6- RTTN*
	oar3_OAR23_7427625	rs404991855	23	7427625	0.40	3.63 × 10^−7^	*RTTN -CD226-DOK6*
	oar3_OAR23_7428353	rs418191944	23	7428353	0.39	4.86 × 10^−6^	*RTTN -CD226-DOK6*
	oar3_OAR23_48239663	rs410842314	23	48239663	0.21	4.09 × 10^−6^	*ZBTB7C-CTIF*

**Table 3:** Information regarding the location and amino acid substitution effect of the identified SNPs according to the Variant Effect Predictor and PolyPhen-2 software in the *NPY* and *CD226* genes. Scores are indicated in brackets. The SNPs are ordered according to their positions in the Oar3.1 genome version (Oar3.1: GenBank acc. Numbers NC_019461 and NC_019480 for *NPY* and *CD226*, respectively).

Gene	dbSNP	Location	Position in OAR Version 3.1	Nucleotide Change	Amino Acid Change	VEP (SIFT Score)	Polyphen-2 (Score)
*NPY*	-	Exon 2	OAR4:g.71593018	G > T	Leu21 = ^1^	-	-
-	rs594346709	Exon 2	OAR4:g.71593068	G > A	Ser18 =	-	-
*CD226*	rs427511555	Exon 2	OAR23:g.7375331	G > A	Thr25 =	-	-
-	rs403900117	Exon 2	OAR23:g.7375377	T > C	Leu41 =	-	-
-	rs588529642	Exon 2	OAR23:g.7375434	A > G	Met60Val	Tolerated (0.1)	Benign (0.03)
-	rs404360094	Exon 3	OAR23:g.7432390	A > G	Asn243Asp	Tolerated (1)	Benign (0.008)

^1^ No amino acid change.

**Table 4:** Type III test for the SNP located at exon 3 of the *CD226* gene on seasonal traits (total days of anoestrous (TDA), oestrous cycling month (OCM) and progesterone cycling month (P4CM)) of Rasa Aragonesa ewes. The least square means (LSMs) and standard errors of alternative genotypes are also shown. Only significant SNPs after Bonferroni correction are shown. Different letters indicate significant differences: **a**, **b**: *p* < 0.05.

Trait	*p*-Value SNP	SNP LSMs		
		AA	AG	GG
TDA	0.0003	120.7 ± 12.21 **a**	69.9 ± 4.88 **b**	78.1 ± 4.94 **b**
P4CM	0.0006	0.64 ± 0.04 **a**	0.83 ± 0.01 **b**	0.79 ± 0.01 **b**
OCM	0.001	0.29 ± 0.05 **a**	0.50 ± 0.02 **b**	0.46 ± 0.02 **b**

**Table 5:** Type III test for the haplotype effects for block 2 of the *CD226* gene using seasonal phenotypic data from Rasa Aragonesa ewes (total days of anoestrous (TDA), oestrous cycling month (OCM) and progesterone cycling month (P4CM)). The least square means (LSMs) and standard errors for the haplotype effect are also shown. Only significant haplotypes after Bonferroni correction are shown. Different letters indicate significant differences: **a**, **b**: *p* < 0.05.

Trait	Haplotype ^1^	Frequency	*p*-Value Haplotype	Haplotype LSMs ^2^		
				0 copies	1 copy	2 copies
TDA	H1(AG)	0.70	0.002	110.3 ± 11.17 **a**	70.7 ± 4.83 **b**	79 ± 5.08 **b**
P4CM	-	-	0.002	0.67 ± 0.04 **a**	0.82 ± 0.01 **b**	0.79 ± 0.01 **b**
OCM	-	-	0.004	0.32 ± 0.04 **a**	0.49 ± 0.02 **b**	0.46 ± 0.02 **b**
TDA	H2(AA)	0.25	0.0007	77.5 ± 4.88 **a**	71.1 ± 4.94 **a**	122.1 ± 12.69 **b**
P4CM	-	-	0.0005	0.79 ± 0.01 **a**	0.83 ± 0.01 **a**	0.63 ± 0.04 **b**
OCM	-	-	0.003	0.46 ± 0.02 **a**	0.49 ± 0.02 **a**	0.29 ± 0.05 **b**

^1^ Block 2 rs588529642–rs404360094. ^2^ 0 copy: LSMs and SE for 0 copies of the haplotype; 1 copy: LSMs and SE for 1 copy of the haplotype; and 2 copies: LSMs and SE for 2 copies of the haplotype.

## Data Availability

The data presented in this study are available on request from the corresponding author.

## Supplementary Materials

The following are available online at https://www.mdpi.com/article/10.3390/ani11041171/s1, Figure S1: Multidimensional scaling (MDS) analysis performed with 188,633 SNPs after pruning in 205 ewes. The analysis showed the 4 clusters of animals included in analysis., Figure S2: Manhattan and Q-Q plots from GWAS analysis for a) TDA, b) P4CM and c) OCM traits in Rasa Aragonesa sheep breed. Chromosomes 1–26 and X (27) are shown. Horizontal green line in the Manhattan plot corresponds to the average threshold value for a FDR of 10% evaluated at the chromosomal level, Table S1: Genome-wide Complex Trait Analysis (GCTA) results for significant Single Nucleotide Polymorphisms (SNPs) at the genome (Bonferroni correction), and chromosome (FDR 0.10) levels for the reproductive seasonality traits studied. Threshold for chromosome level (FDR 0.10) was indicated (pval_FDR10), Table S2: Genotypic and allelic frequencies of the genotyped SNPs for the validation studies, Table S3: Type III test for the body condition score (BCS), live weight (LW), the age (A), and SNP effects for the *CD226* and *NPY* polymorphisms using the seasonality phenotype data from Rasa Aragonesa ewes. The least square means (LSMs) and standard errors are also shown. Different letters indicate significant differences: a, b: *p* < 0.05 after Bonferroni correction, Table S4: Haplotypes combination and frequency block 1 (OAR4:g.71593018- rs594346709) and for block 2 (rs588529642–rs404360094). Only haplotypes with a frequency higher than 1% are shown, Table S5: Block 1 Type III test for the body condition (BC), live weight (LW), the age (A), and Haplotype (H) for the *NPY* polymorphisms using the seasonality phenotype data from Rasa Aragonesa ewes. The least square means (LSMs) and standard are also shown. Different letters indicate significant differences: a, b: *p* < 0.05 after Bonferroni correction, Table S6: Block 2 Type III test for the body condition (BC), live weight (LW), the age (A), and Haplotype (H) for the *CD226* polymorphisms using the seasonality phenotype data from Rasa Aragonesa ewes. The least square means (LSMs) and standard are also shown. Different letters indicate significant differences: a, b: *p* < 0.05 after Bonferroni correction.
