# Supplementary material for: Genome-Wide Association Study Demonstrates the Role Played by the CD226 Gene in Rasa Aragonesa Sheep Reproductive Seasonality
Source: Animals (Basel). 2021 Apr 19;11(4):1171. doi: 10.3390/ani11041171 (PMC8074133; doi:10.3390/ani11041171)
Supplement: Supplementary file 1 [file animals-11-01171-s001.zip › Figure S1.pdf]

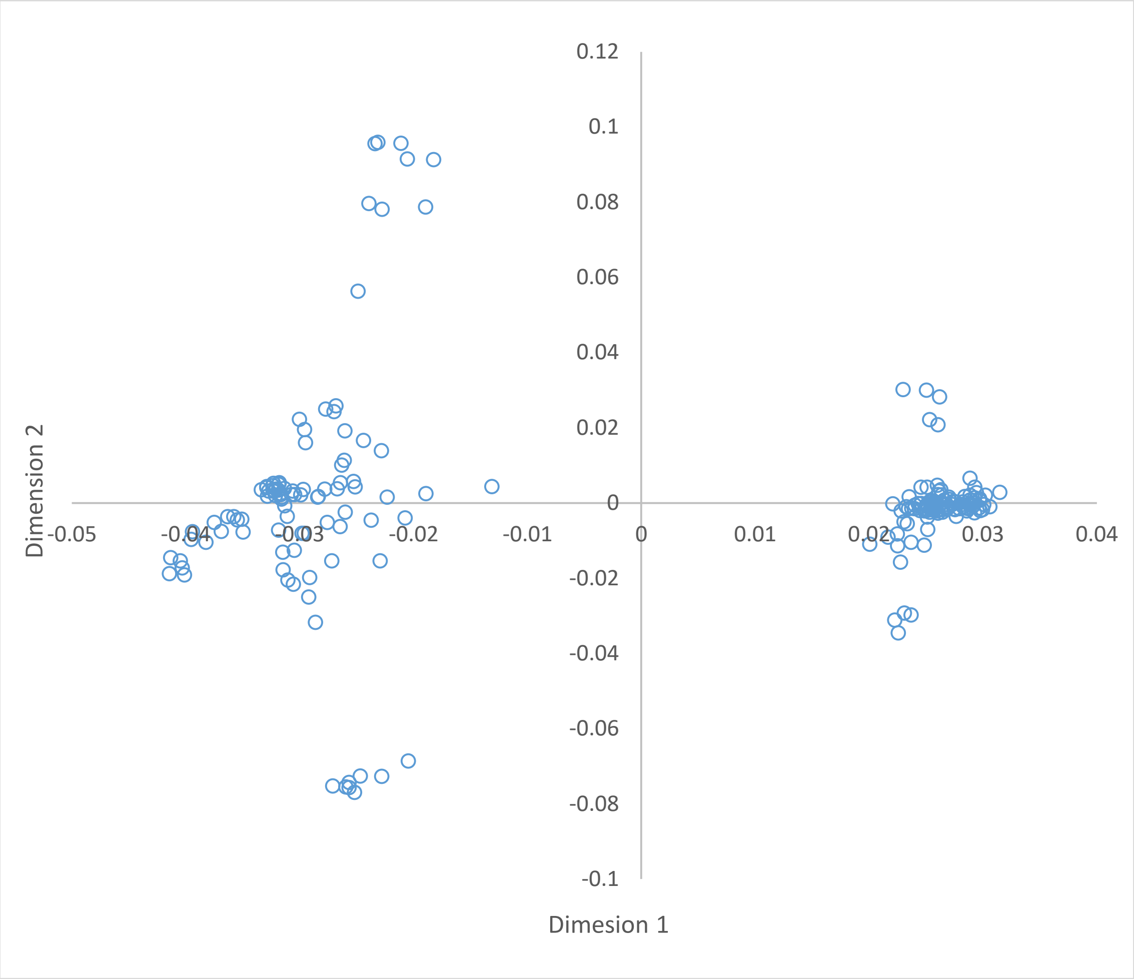

Figure S1. Multidimensional scaling (MDS) analysis performed with 188,633 SNPs after pruning in 205 ewes. The analysis showed the 4 clusters of animals included in analysis.
