# Supplementary material for: Genome-Wide Association Study Demonstrates the Role Played by the CD226 Gene in Rasa Aragonesa Sheep Reproductive Seasonality
Source: Animals (Basel). 2021 Apr 19;11(4):1171. doi: 10.3390/ani11041171 (PMC8074133; doi:10.3390/ani11041171)
Supplement: Supplementary file 1 [file animals-11-01171-s001.zip › Figure S2.pdf]

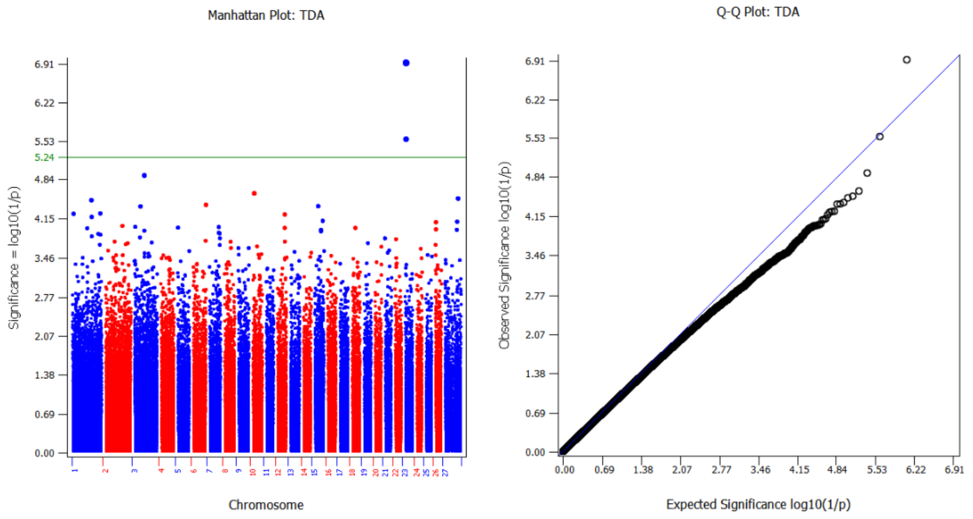

(a)

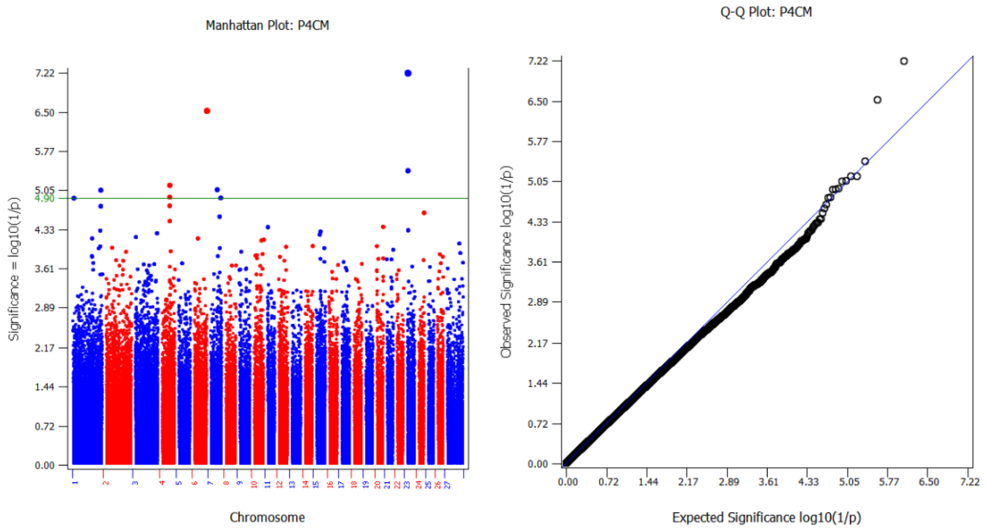

(b)

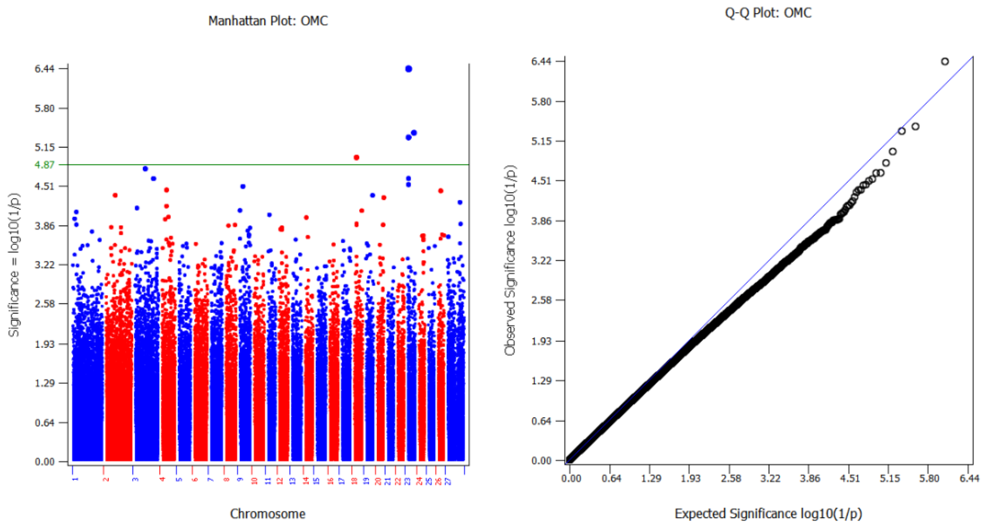

(c)

Figure S2. Manhattan and Q-Q plots from GWAS analysis for (a) TDA, (b) P4CM and (c) OCM traits in Rasa Aragonesa sheep breed. Chromosomes 1-26 and X (27) are shown. Horizontal green line in the Manhattan plot corresponds to the average threshold value for a FDR of 10% evaluated at the chromosomal level.
