# Supplementary material for: Genome-Wide Association Study Demonstrates the Role Played by the CD226 Gene in Rasa Aragonesa Sheep Reproductive Seasonality
Source: Animals (Basel). 2021 Apr 19;11(4):1171. doi: 10.3390/ani11041171 (PMC8074133; doi:10.3390/ani11041171)
Supplement: Supplementary file 1 [file animals-11-01171-s001.zip › Table S1.docx]

**Table S1**. Genome-wide Complex Trait Analysis (GCTA) results for significant Single Nucleotide Polymorphisms (SNPs) at the genome (Bonferroni correction), and chromosome (FDR 0.10) levels for the reproductive seasonality traits studied. Threshold for chromosome level (FDR 0.10) was indicated (pval_FDR10).

| Trait | Chr | SNP | bp | A1 | A2 | Freq | b | se | p | pval_BH | pval_Bonf | qval | pval_FDR10 |
| --- | --- | --- | --- | --- | --- | --- | --- | --- | --- | --- | --- | --- | --- |
| TDA | 23 | oar3_OAR23_7427625 | 7427625 | A | G | 0.40 | -28.35 | 5.36 | 1.22E-07 | 0.07 | 0.07 | 0.07 | 1.40E-05 |
|  | 23 | oar3_OAR23_7428353 | 7428353 | A | G | 0.39 | -25.04 | 5.34 | 2.77E-06 | 0.81 | 1 | 0.81 | 1.40E-05 |
| P4CM | 23 | oar3_OAR23_7427625 | 7427625 | A | G | 0.40 | 0.11 | 0.02 | 6.07E-08 | 0.04 | 0.04 | 0.04 | 1.40E-05 |
|  | 6 | oar3_OAR6_114690755 | 114690755 | G | A | 0.22 | -0.11 | 0.02 | 3.01E-07 | 0.09 | 0.18 | 0.09 | 3.80E-06 |
|  | 23 | oar3_OAR23_7428353 | 7428353 | A | G | 0.39 | 0.09 | 0.02 | 3.83E-06 | 0.72 | 1 | 0.72 | 1.40E-05 |
|  | 4 | oar3_OAR4_71540823 | 71540823 | A | G | 0.22 | -0.10 | 0.02 | 7.12E-06 | 0.72 | 1 | 0.72 | 7.40E-06 |
|  | 4 | oar3_OAR4_71552651 | 71552651 | G | A | 0.22 | -0.10 | 0.02 | 7.12E-06 | 0.72 | 1 | 0.72 | 7.40E-06 |
|  | 7 | oar3_OAR7_57807908 | 57807908 | G | A | 0.04 | -0.21 | 0.05 | 8.59E-06 | 0.72 | 1 | 0.72 | 1.30E-05 |
|  | 7 | oar3_OAR7_87670575 | 87670575 | A | G | 0.12 | -0.12 | 0.03 | 1.22E-05 | 0.72 | 1 | 0.72 | 1.30E-05 |
| OCM | 23 | oar3_OAR23_7427625 | 7427625 | A | G | 0.40 | 0.12 | 0.02 | 3.63E-07 | 0.21 | 0.21 | 0.21 | 2.90E-05 |
|  | 23 | oar3_OAR23_48239663 | 48239663 | A | C | 0.21 | -0.12 | 0.03 | 4.09E-06 | 0.95 | 1 | 0.95 | 2.90E-05 |
|  | 23 | oar3_OAR23_7428353 | 7428353 | A | G | 0.39 | 0.11 | 0.02 | 4.86E-06 | 0.95 | 1 | 0.95 | 2.90E-05 |
|  | 23 | oar3_OAR23_6962033 | 6962033 | A | G | 0.09 | 0.17 | 0.04 | 2.30E-05 | 1 | 1 | 1 | 2.90E-05 |
