# Supplementary material for: Genome-Wide Association Study Demonstrates the Role Played by the CD226 Gene in Rasa Aragonesa Sheep Reproductive Seasonality
Source: Animals (Basel). 2021 Apr 19;11(4):1171. doi: 10.3390/ani11041171 (PMC8074133; doi:10.3390/ani11041171)
Supplement: Supplementary file 1 [file animals-11-01171-s001.zip › Table S2.docx]

**Table S2.** Genotypic and allelic frequencies of the genotyped SNPs for the validation studies

| **Gene** | **Location** | **Position in Oar 3.1** | **dbSNPs** | **Genotype** | **Genotype frequencies** | **Allele** | **Allele frequencies** |
| --- | --- | --- | --- | --- | --- | --- | --- |
| NPY | Exon 2 | OAR4:g.71593018 | - | GG | 0.953 | G | 0.975 |
|  |  |  |  | GT | 0.047 | T | 0.025 |
|  |  | OAR4:g.71593068 | rs594346709 | AA | 0.004 | G | 0.918 |
|  |  |  |  | AG | 0.158 | A | 0.082 |
|  |  |  |  | GG | 0.838 |  |  |
| CD226 | Exon 2 | OAR23:g.7375434 | rs588529642 | AA | 0.907 | A | 0.958 |
|  |  |  |  | AG | 0.093 | G | 0.042 |
|  | Exon 3 | OAR23:g.7375434 | rs404360094 | AA | 0.047 | G | 0.728 |
|  |  |  |  | AG | 0.453 | A | 0.272 |
|  |  |  |  | GG | 0.500 |  |  |
