# Supplementary material for: Genome-Wide Association Study Demonstrates the Role Played by the CD226 Gene in Rasa Aragonesa Sheep Reproductive Seasonality
Source: Animals (Basel). 2021 Apr 19;11(4):1171. doi: 10.3390/ani11041171 (PMC8074133; doi:10.3390/ani11041171)
Supplement: Supplementary file 1 [file animals-11-01171-s001.zip › Table S3.docx]

**Table S3.** Type III test for the body condition score (BCS), live weight (LW), the age (A), and SNP effects for the *CD226* and *NPY* polymorphisms using the seasonality phenotype data from Rasa Aragonesa ewes. The least square means (LSMs) and standard errors are also shown. Different letters indicate significant differences: a, b: P < 0.05 after Bonferroni correction.

| **SNP** |  |  |  | **P Value** | | | |  | **LSMs SNP** | | |
| --- | --- | --- | --- | --- | --- | --- | --- | --- | --- | --- | --- |
|  |  | **Phenotype** |  | **BCS** | **LW** | **A** | **SNP** |  |  |  |  |
|  |  |  |  |  |  |  |  |  |  |  |  |
| **rs588529642** |  |  |  |  |  |  |  |  | **AA** | **AG** | **GG** |
|  |  | TDA |  | 0.346 | 0.005 | 0.100 | 0.602 |  | 77.8 ± 4.09 | 72.5 ± 10.48 | - |
|  |  | P4CM |  | 0.096 | 0.065 | 0.426 | 0.733 |  | 0.80 ± 0.01 | 0.81 ± 0.04 | - |
|  |  | OCM |  | 0.118 | 0.018 | 0.013 | 0.643 |  | 0.46 ± 0.01 | 0.48 ± 0.04 | - |
| **rs404360094** |  |  |  |  |  |  |  |  | **AA** | **AG** | **GG** |
|  |  | TDA |  | 0.367 | 0.001 | 0.149 | 0.0003 |  | 120.7 ± 12.21**a** | 69.9 ± 4.88**b** | 78.1 ± 4.94**b** |
|  |  | P4CM |  | 0.092 | 0.035 | 0.517 | 0.0006 |  | 0.64 ± 0.04**a** | 0.83 ± 0.01**b** | 0.79 ± 0.01**b** |
|  |  | OCM |  | 0.118 | 0.008 | 0.018 | 0.001 |  | 0.29 ± 0.05**a** | 0.50 ± 0.02**b** | 0.46 ± 0.02**b** |
| **rs594346709** |  |  |  |  |  |  |  |  | **AA** | **AG** | **GG** |
|  |  | TDA |  | 0.435 | 0.004 | 0.089 | 0.853 |  | 55.4 ± 44.94 | 79.5 ± 7.42 | 77.5 ± 4.28 |
|  |  | P4CM |  | 0.116 | 0.061 | 0.383 | 0.629 |  | 0.96 ± 0.17 | 0.79 ± 0.02 | 0.80 ± 0.01 |
|  |  | OCM |  | 0.108 | 0.022 | 0.012 | 0.658 |  | 0.43 ± 0.20 | 0.49 ± 0.03 | 0.46 ± 0.01 |
| **OAR4:g.71593018** |  |  |  |  |  |  |  |  | **GG** | **GT** | **TT** |
|  |  | TDA |  | 0.414 | 0.005 | 0.077 | 0.815 |  | 78.1 ± 4.11 | 75.2 ± 12.55 | - |
|  |  | P4CM |  | 0.106 | 0.074 | 0.340 | 0.809 |  | 0.80 ± 0.01 | 0.81 ± 0.04 | - |
|  |  | OCM |  | 0.105 | 0.015 | 0.015 | 0.884 |  | 0.46 ± 0.01 | 0.47 ± 0.05 | - |
