# Supplementary material for: Genome-Wide Association Study Demonstrates the Role Played by the CD226 Gene in Rasa Aragonesa Sheep Reproductive Seasonality
Source: Animals (Basel). 2021 Apr 19;11(4):1171. doi: 10.3390/ani11041171 (PMC8074133; doi:10.3390/ani11041171)
Supplement: Supplementary file 1 [file animals-11-01171-s001.zip › Table S4.docx]

**Table S4**. Haplotypes combination and frequency block 1 (OAR4:g.71593018- rs594346709) and for block 2 (rs588529642 - rs404360094). Only haplotypes with a frequency higher than 1% are shown.

|  | **Haplotype** | **Combination** | **Frequency** |
| --- | --- | --- | --- |
| Block 1 | h1 | GG | 0.893 |
|  | h2 | GA | 0.082 |
|  | h3 | TG | 0.025 |
| Block 2 | h1 | AG | 0.71 |
|  | h2 | AA | 0.25 |
|  | h3 | GA | 0.02 |
