# Supplementary material for: Genome-Wide Association Study Demonstrates the Role Played by the CD226 Gene in Rasa Aragonesa Sheep Reproductive Seasonality
Source: Animals (Basel). 2021 Apr 19;11(4):1171. doi: 10.3390/ani11041171 (PMC8074133; doi:10.3390/ani11041171)
Supplement: Supplementary file 1 [file animals-11-01171-s001.zip › Table S5.docx]

**Table S5.** Block 1 Type III test for the body condition (BC), live weight (LW), the age (A), and Haplotype (H) for the NPY polymorphisms using the seasonality phenotype data from Rasa Aragonesa ewes. The least square means (LSMs) and standard are also shown. Different letters indicate significant differences: a, b: P < 0.05 after Bonferroni correction.

| **H** |  |  |  | **P Value** | | | |  | **Haplotype LSMs** | | |
| --- | --- | --- | --- | --- | --- | --- | --- | --- | --- | --- | --- |
|  |  | **Phenotype** |  | **BC** | **LW** | **A** | **H** |  |  |  |  |
|  |  |  |  |  |  |  |  |  |  |  |  |
| **H1** |  |  |  |  |  |  |  |  | **0** copies | **1** copy | **2** copies |
|  |  | TDA |  | 0.362 | 0.006 | 0.089 | 0.335 |  | 50.6 ± 18.68 | 78.6 ± 6.66 | 77.7 ± 4.41 |
|  |  | P4CM |  | 0.087 | 0.073 | 0.399 | 0.307 |  | 0.91 ± 0.07 | 0.79 ± 0.02 | 0.80 ± 0.01 |
|  |  | OCM |  | 0.087 | 0.024 | 0.011 | 0.273 |  | 0.58 ± 0.08 | 0.48 ± 0.02 | 0.46 ± 0.01 |
| **H2** |  |  |  |  |  |  |  |  |  |  |  |
|  |  | TDA |  | 0.439 | 0.004 | 0.084 | 0.845 |  | 77.1 ± 4.29 | 79.5 ± 7.47 | 55.4 ± 45.18 |
|  |  | P4CM |  | 0.120 | 0.057 | 0.399 | 0.624 |  | 0.80 ± 0.01 | 0.79 ± 0.02 | 0.96 ± 0.17 |
|  |  | OCM |  | 0.105 | 0.023 | 0.010 | 0.691 |  | 0.46 ± 0.01 | 0.49 ± 0.03 | 0.43 ± 0.20 |
| **H3** |  |  |  |  |  |  |  |  |  |  |  |
|  |  | TDA |  | 0.428 | 0.005 | 0.070 | 0.840 |  | 77.7 ± 4.12 | 75.1 ± 12.60 | - |
|  |  | P4CM |  | 0.112 | 0.070 | 0.344 | 0.832 |  | 0.80 ± 0.01 | 0.81 ± 0.04 | - |
|  |  | OCM |  | 0.108 | 0.016 | 0.011 | 0.911 |  | 0.46 ± 0.01 | 0.47 ± 0.05 | - |
