# Supplementary material for: Genome-Wide Association Study Demonstrates the Role Played by the CD226 Gene in Rasa Aragonesa Sheep Reproductive Seasonality
Source: Animals (Basel). 2021 Apr 19;11(4):1171. doi: 10.3390/ani11041171 (PMC8074133; doi:10.3390/ani11041171)
Supplement: Supplementary file 1 [file animals-11-01171-s001.zip › Table S6.docx]

**Table S6.** Block 2 Type III test for the body condition (BC), live weight (LW), the age (A), and Haplotype (H) for the *CD226* polymorphisms using the seasonality phenotype data from Rasa Aragonesa ewes. The least square means (LSMs) and standard are also shown. Different letters indicate significant differences: a, b: P < 0.05 after Bonferroni correction.

| **H** |  |  |  | **P Value** | | | |  | **Haplotype LSMs** | | |
| --- | --- | --- | --- | --- | --- | --- | --- | --- | --- | --- | --- |
|  |  | **Phenotype** |  | **BC** | **LW** | **A** | **H** |  |  |  |  |
|  |  |  |  |  |  |  |  |  |  |  |  |
| **H1** |  |  |  |  |  |  |  |  | **0** copies | **1** copy | **2** copies |
|  |  | **TDA** |  | 0.488 | 0.002 | 0.109 | 0.002 |  | 110.3 ± 11.17**a** | 70.7 ± 4.83**b** | 79 ± 5.08**b** |
|  |  | **P4CM** |  | 0.131 | 0.040 | 0.453 | 0.002 |  | 0.67 ± 0.04**a** | 0.82 ± 0.01**b** | 0.79 ± 0.01**b** |
|  |  | **OCM** |  | 0.127 | 0.008 | 0.016 | 0.004 |  | 0.32 ± 0.04**a** | 0.49 ± 0.02**b** | 0.46 ± 0.02**b** |
| **H2** |  |  |  |  |  |  |  |  |  |  |  |
|  |  | **TDA** |  | 0.414 | 0.002 | 0.107 | 0.0007 |  | 77.5 ± 4.88**a** | 71.1 ± 4.94a | 122.1 ± 12.69**b** |
|  |  | **P4CM** |  | 0.098 | 0.046 | 0.402 | 0.0005 |  | 0.79 ± 0.01**a** | 0.83 ± 0.01a | 0.63 ± 0.04**b** |
|  |  | **OCM** |  | 0.098 | 0.010 | 0.017 | 0.003 |  | 0.46 ± 0.02**a** | 0.49 ± 0.02a | 0.29 ± 0.05**b** |
| **H3** |  |  |  |  |  |  |  |  |  |  |  |
|  |  | **TDA** |  | 0.422 | 0.005 | 0.085 | 0.553 |  | 77.7 ± 4.05 | 69.7 ± 13.72 | - |
|  |  | **P4CM** |  | 0.110 | 0.067 | 0.382 | 0.701 |  | 0.80 ± 0.01 | 0.82 ± 0.05 | - |
|  |  | **OCM** |  | 0.103 | 0.015 | 0.014 | 0.240 |  | 0.46 ± 0.01 | 0.53 ± 0.06 | - |
